# The impact of door-to-electrocardiogram time on door-to-balloon time after achieving the guideline-recommended target rate

**DOI:** 10.1371/journal.pone.0222019

**Published:** 2019-09-09

**Authors:** Chih-Kuo Lee, Shih-Wei Meng, Ming-Hsien Lee, Hsiu-Chi Chen, Chia-Ling Wang, Hui-Ning Wang, Min-Tsun Liao, Mu-Yang Hsieh, Yung-Chung Huang, Edward Pei-Chuan Huang, Chih-Cheng Wu

**Affiliations:** 1 Department of Internal Medicine, National Taiwan University Hospital Hsin-Chu Branch, Hsinchu, Taiwan; 2 Department of Nursing, National Taiwan University Hospital, Hsin-Chu Branch, Hsinchu, Taiwan; 3 Quality Control Center, National Taiwan University Hospital, Hsin-Chu Branch, Hsinchu, Taiwan; 4 College of Medicine, National Taiwan University, Taipei, Taiwan; 5 Department of Emergency, Taipei City Hospital, Renai Branch, Taipei, Taiwan; 6 Department of Emergency Medicine, National Taiwan University Hospital Hsin-Chu Branch, Hsinchu, Taiwan; 7 Cardiovascular Center, National Taiwan University Hospital Hsin-Chu Branch, Hsinchu, Taiwan; 8 Institute of Biomedical Engineering, National Tsing-Hwa University, Hsinchu, Taiwan; 9 Institute of Cellular and System Medicine, National Health Research Institute, Miaoli, Taiwan; Duke-NUS Medical School, SINGAPORE

## Abstract

**Background:**

Little is known about the components and contributing factors of door-to-balloon time after implementation of Door-to-Balloon Alliance quality-improving (QI) strategies, including the impact of door-to-ECG time on door-to-balloon time.

**Objective:**

We investigated whether modification of emergency department (ED) triage processes could improve door-to-ECG and door-to-balloon times after implementation of QI strategies.

**Methods:**

This was a retrospective before-and-after study of a prospectively collected database. From June 2014 to October 2014, interventions were implemented in our ED, including a protocol-driven ECG initiation and moving an ECG station and technician to the triage area. The primary outcome was the percentage of patients with ST-elevation myocardial infarction (STEMI) who received ECG within 10 min of arrival; the secondary outcome was the percentage of patients with door-to-balloon times of <90 min from arrival. Patients from the year pre- and post-QI initiative were defined as the control and intervention groups, respectively.

**Results:**

Enrollment comprised 214 patients with STEMI: 109 before the intervention and 105 after the intervention. We analyzed the components of the door-to-balloon process and found the door-to-ECG process was the most critical interval of delay (20.8%). Unrecognized symptoms were the most common cause of delay in the door-to-ECG process resulting in a significant impact on the door-to-balloon time. The intervention group had a higher percentage of patients with door-to-ECG times <10 min than did the control group (93.3% vs. 79.8%, *p* = 0.005), with a corresponding improvement in door-to-balloon times <90 min (91.1% vs. 76.2%, *p* = 0.007). In subgroup analysis, the intervention benefits occurred only in non-transferred or walk-in patients. After adjustment for possible co-variates, the QI interventions remained a significant contributing factor for achieving the door-to-ECG and door-to-balloon targets.

**Conclusions:**

The modification of ED triage processes through implementation of QI strategies are effective in achieving better door-to-ECG times and thus, achieving door-to-balloon times <90 min. In patients presenting with ambiguous symptoms, improved door-to ECG target achievement rates, through a protocol-driven and multidisciplinary approach allows for earlier identification of STEMI.

## Introduction

National guidelines recommend that, if immediately available, primary percutaneous coronary intervention (PCI) should be performed in patients with ST elevation myocardial infarction (STEMI) [1,2]. The time from the patient’s arrival at hospital to reperfusion is strongly associated with the morbidity and mortality of patients with STEMI. Therefore, a 90 min target for door-to-balloon time is generally recommended as the most critical quality measure of hospital performance in acute coronary care [3]. In 2006, Bradly et al. identified 6 key strategies for reducing door-to-balloon time, which included having a single call to a page operator to activate the catheterization (cath) lab, having the emergency department (ED) activate the cath lab, pre-hospital cath lab activation, expecting cath lab staff to arrive within 20 min of being paged, having an attending cardiologist on site at all times, and having staff in the ED and cath lab use real-time feedback [4]. Later, the Door-to-Balloon Alliance suggested adapting these strategies to shorten the door-to-balloon times [5]. Despite the improvements from the Door-to-Balloon Alliance, a substantial portion of STEMI patients’ door-to-balloon times still exceeded the 90 min target [6–9]. Although further reduction of the door-to-balloon time below the 90 min target showed no mortality benefit, continuous efforts to minimize the outliers that fall above the 90 min target should be emphasized [10].

Although door-to-balloon times are decreasing, current strategies focus on the process after a STEMI diagnosis is established. Rapid recognition of STEMI is the first step in achieving a timely reperfusion. The diagnosis of STEMI is based on a combination of electrocardiogram (ECG) findings, clinical history, and cardiac enzyme levels. Early acquisition of ECGs in the ED plays a central role in the decision for reperfusion therapy. Therefore, a 10 min target for door-to-ECG time is recommended in the majority of national guidelines [1, 2, 11]. Nonetheless, several studies have shown that only one-third of patients with acute coronary syndrome (ACS) achieved the ECG acquisition target of 10 min. Although societies have made suggestions for performing ECG in the ED, only a minority of the literature addresses how to adhere to the 10 min goal [12–15]. In addition, it is not clear how great the impact of door-to-ECG time is, especially after the implantation of best-practice techniques.

Accordingly, the aims of this study were to evaluate whether changes in triage and ECG processes could minimize the outliers of the door-to-ECG time target and to determine the impact of door-to-ECG time on the door-to-balloon process.

## Materials and methods

### Study design and setting

This was a retrospective before-and-after study of a prospectively collected database from an urban teaching hospital with an annual census of approximately 80,000 ED visits. Our government audited the quality of care for ACS in 3 year intervals, according to the regulations for emergency-care-capacity-accreditation (ECCA). Four quality indicators were defined, including the target rates of door-to-ECG time <10 min, door-to-balloon time <90 min or door-to-needle time <30 min, door-to-cardiac enzyme time <120 min, and prescription of dual antiplatelet agents. These quality indicators had been collected by our quality control center since 2005 with a monthly analysis of cases that did not fulfill any 1 of the 4 quality indicators. From the first ECCA audit in 2008, the best practice strategies suggested by the door-to-balloon Alliance were implemented gradually, including ED activation, single call to central page, staff arrival within 20 minutes, cardiologist on site, real-time feedback, team approach, and action plans [4,16]. In 2014, facing the coming audit by the ECCA and variations in the rate of meeting the door-to-ECG target time, a working group composed of cardiology, emergency medicine, nursing, and quality control center specialists was formed. According to the feedback from the previous audit, the primary goal of the initiative was to improve the rate of meeting the door-to-ECG target time. To achieve this goal, a series of interventions were implemented from June 2014 to October 2014. From the database, we extracted patients with a discharge diagnosis of STEMI within 1 year before and 1 year after the quality-improving initiative.

### Ethics statement

The study was approved by the Institutional Review Board of the National Taiwan University Hospital, Hsin-Chu Branch. All data applied from the quality control center were fully anonymized and our Institutional Review Board waived the requirement for informed consent.

### Before intervention

**Fig 1:**
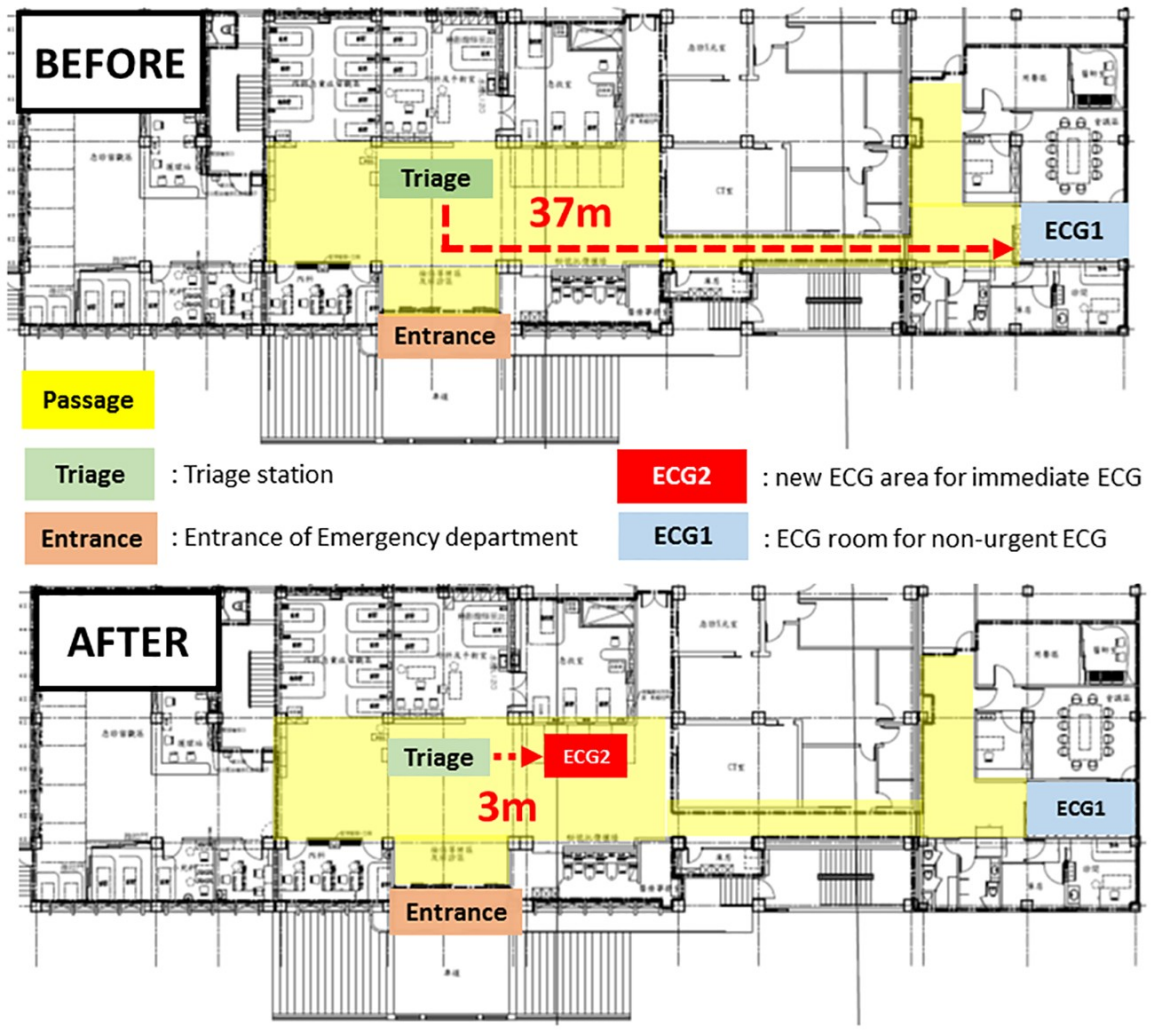
Layout of the emergency department with marks of the locations for ECG examination before and after quality-improving initiatives.

Before the intervention, when a patient came to our ED, the patient would be directed to a triage area for a brief registration that included the chief complaint. The triage nurse would make the decision whether to perform an ECG immediately, depending on the chief complaint and the general appearance of the patient. If an immediate ECG was indicated, the patient would be directed to the ECG room in the ED, about 37 meters from the triage area (Fig 1). Unstable patients were escorted to the treatment area where an ECG would be performed at the bedside.

### Intervention

The interventions included 4 components, implemented step by step. Firstly, pre-defined criteria for initiating an ECG immediately were set up at the triage station, including chest pain, chest tightness, shortness of breath, upper abdominal discomfort (if more than 40 years old), syncope, and hemodynamic instability (systolic blood pressure less than 90 mmHg or heart rate less than 50/min). When either one of these criteria were found during the triage process, the triage nurse would initiate an ECG examination immediately. Secondly, one of the two ECG machines in ED was moved to a designated area just beside the triage area to shorten the transportation time (Fig 1). In cases that fulfilled the immediate ECG criteria, a triage nurse would page an ECG technician to perform the ECG examination using the machine near the triage area. Thirdly, a technician was assigned to be on duty and carried a cell phone to respond to immediate ECG activation. The technician’s primary responsibility was performing ECGs initiated by the triage nurse and delivering them to attending physicians, with priority over other ED duties. Fourthly, the triage nurse would assist in preparing the patient for the ECG process before the arrival of the ECG technician. All triage nurses had to undergo training of ECG examination before they could participate in the ECG examination. These measures were introduced step by step and fully implemented by November 2014.

### After intervention

**Fig 2:**
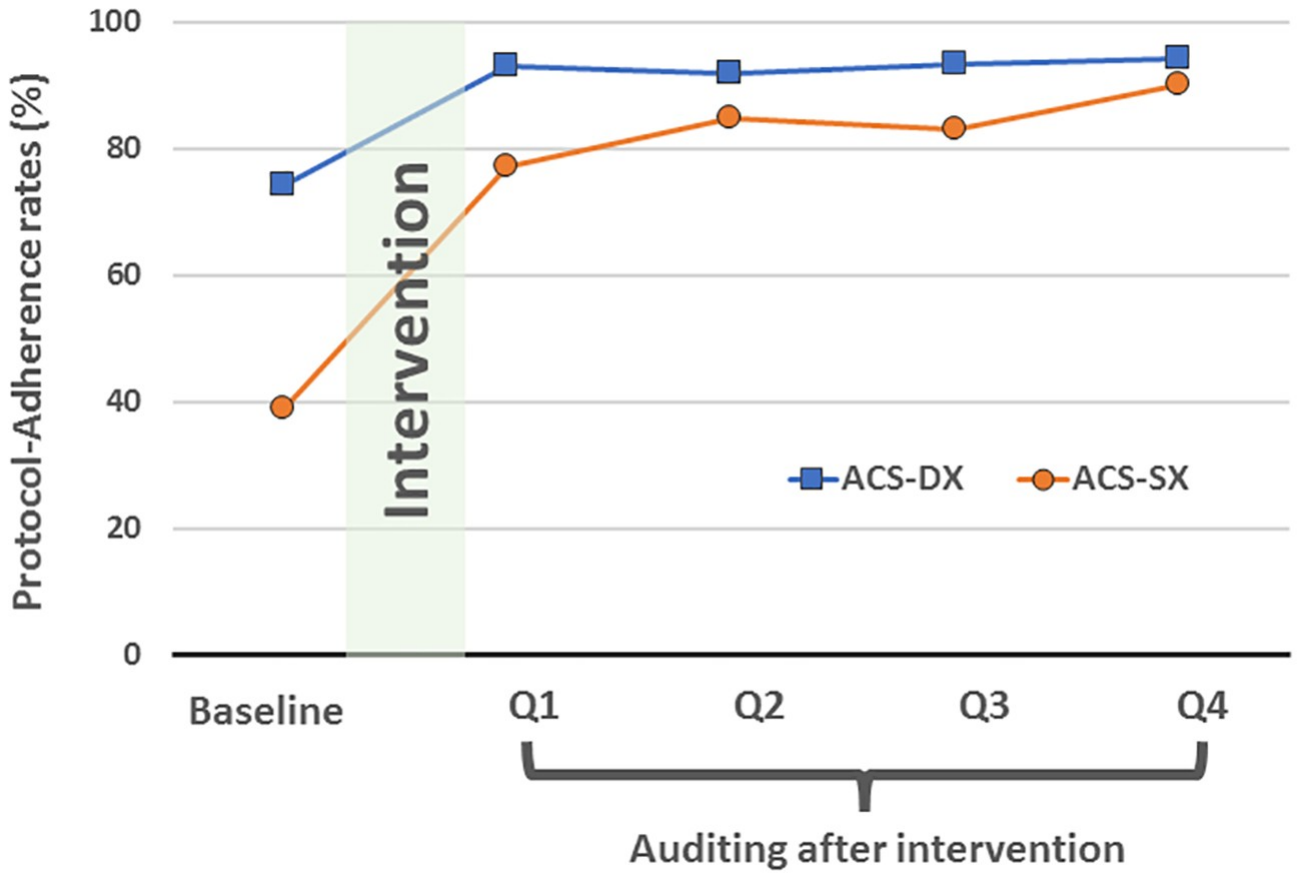
Protocol-adherence rate of immediate ECG initiation at baseline and quarterly reports (Q1, Q2, Q3, Q4) after quality-improving initiatives. Protocol adherence rate was defined as patients with ECG immediately initiated divided by patients fulfilling immediate-ECG initiation criteria. Blue square (**ACS-DX)**: Protocol-adherence rate of patients with a diagnosis of acute coronary syndrome at discharge; Orange circle (**ACS-SX)**: Protocol-adherence rate of patients with symptom(s) fulfilling immediate-ECG initiation criteria at the triage station.

After intervention, the implementation of these measures was reviewed in the monthly performance meetings. The door-to-ECG time of all patients with a discharge diagnosis of ACS was monitored by our quality control center. Causes of delay were analyzed for all outliers. The reports of audits were fed back to the responsible personnel and their group leaders. The fidelity of protocol-driven ECG initiation was measured by the protocol-adherence rate of patients who fulfill the pre-specified criteria of immediate ECG initiation. The protocol-adherence rates were prospectively monitored for all patients with a discharge diagnosis of ACS. They were also retrospectively audited by selecting patients who visited the ED on the first day of a month. The results of fidelity measurement by protocol-adherence rates are provided in Fig 2.

### Data collection and outcomes

We arbitrarily choose patients within 1 year before the quality-improving initiative was implemented (June 2013 to May 2014) as the control group and patients within 1 year after the initiative was implemented (November 2014 to October 2015) as the intervention group. The data were extracted from the prospectively-collected ACS database from the quality control center of our hospital. In the database, the arrival time was extracted from the Electronic Medical Record System (EMRS). The ECG time was extracted from the electronic copy of the ECG stored in the Picture Archiving and Communication System (PACS). The balloon time was recorded from the angiographic image of thrombus aspiration or balloon dilatation in the PACS system. The date and time settings of the ECG machines in the ED and the hemodynamic recording system in the catheterization laboratory were synchronized to the computer-based health information system every day. All the data captured were double-checked by the case manager in the quality control center. The primary outcome for this study was the rate of meeting the door-to-ECG target time of <10 min and the secondary outcome was the rate of meeting the door-to-balloon target time of <90 min.

### Statistical analysis

Data are presented as means (standard deviation, SD) for normally distributed variables and medians (interquartile range, IQR) for non-normally distributed variables. Comparisons of continuous variables were made using the Student’s *t*-test or the Mann-Whitney *U* test as appropriate, and comparisons of proportions were made using the chi-squared test. We assumed the baseline door-to-ECG target rate to be 70%, and a 20% improvement would be achieved after the quality initiative. The required sample size for analysis was 62 patients in each group with 80% power and a type 1 error of 0.05. Because the number of STEMI patients was around 100 patients per year in our hospital, the interval of enrollment was arbitrarily selected as 1 year before and after intervention to balance the seasonal effect. To adjust for possible confounders, relative risk of door-to-ECG <10 min and door-to-balloon <90 min was analyzed using logistic regression. All the analyses were performed using SPSS statistical software version 21.0 (IBM Corp., Armonk, NY USA). A *p* value <0.05 was considered to be statistically significant.

## Results

### Characteristics of study participants

**Fig 3:**
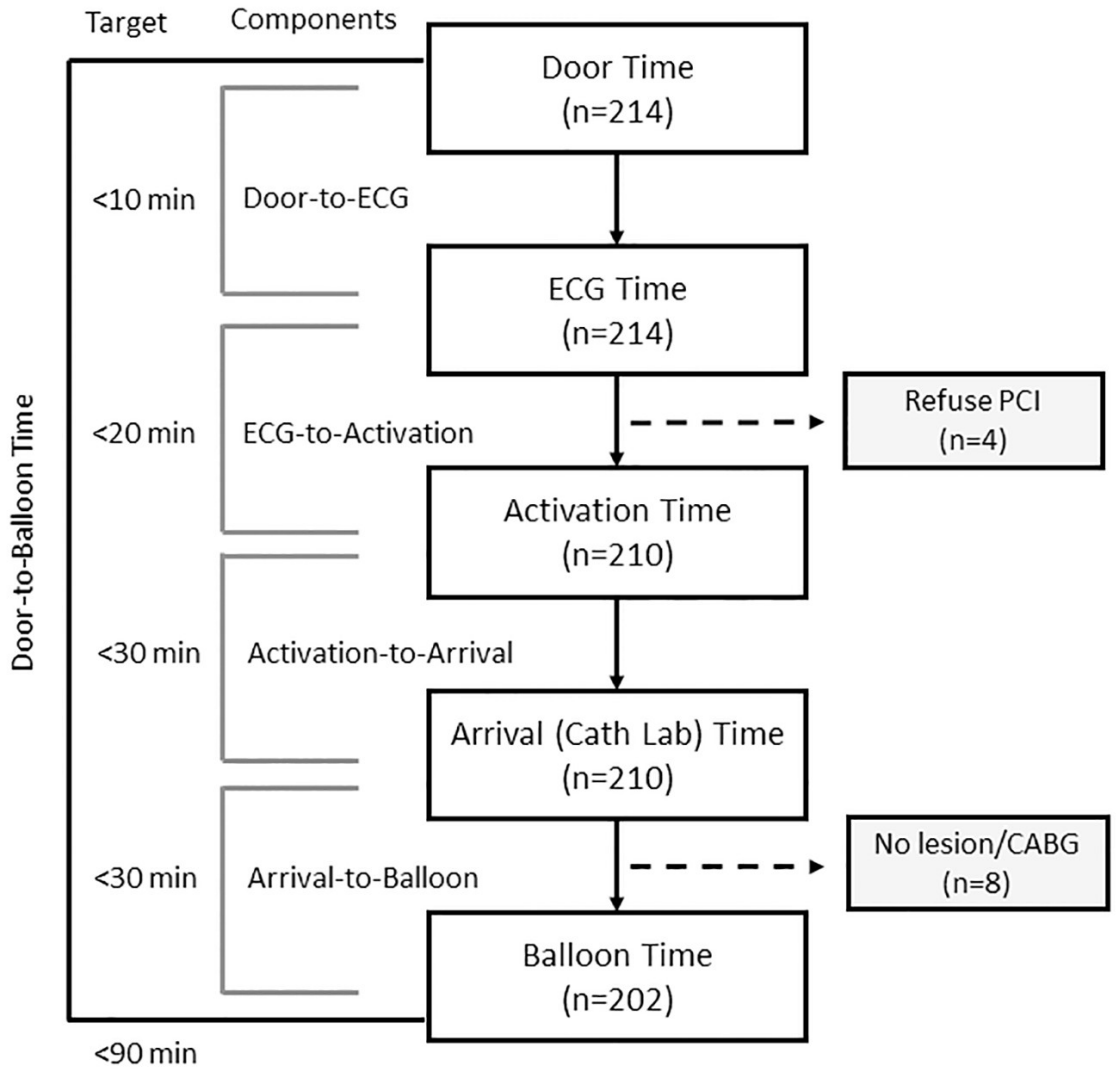
Schematic representation of the breakdown of door-to-balloon time into components with targets. (Abbreviations: PCI, percutaneous coronary intervention; CABG, coronary artery bypass graft surgery).

In the study period, 214 patients with STEMI were enrolled: 109 patients before the intervention and 105 patients after the intervention (Table 1). Their mean age was 62 (SD, 15) years and 163 (76%) of them were male. One hundred and two patients (48%) arrived in the ED on their own and 112 patients (52%) arrived in the ED by ambulance. One hundred and sixteen patients (54%) were transferred from another hospital. Sixty-three patients (29%) presented as acute during daytime and 151 patients (71%) arrived at the ED during the night. Primary PCI was not performed in 12 patients due to the following reasons: terminal stage (n = 1), refusal by patient (n = 3), unsuitable coronary anatomy (n = 5), and insignificant stenosis (n = 3) (Fig 3). Patients in the control and intervention groups were comparable regarding their age, sex, risk factors, history of coronary artery disease, instability, and mode of arrival (Table 1).

**Table 1:** Baseline characteristics of study participants before and after interventions.

Factors	Before (N = 109)	After (N = 105)	*p-value*
**Age (yrs.)**	63 (14)	61 (15)	0.303
**Male/female**	77/32	86/19	0.056
**Risk factors**			
Hypertension	64 (63%)	64 (61%)	0.779
Diabetes	41 (38%)	40 (38%)	0.999
Smoker	41 (38%)	38 (36%)	0.888
Hyperlipidemia	43 (39%)	51 (49%)	0.215
Family history	5 (5%)	4 (4%)	0.999
**CAD history**	12 (11%)	19 (18%)	0.175
**Unstable vital signs**	18 (17%)	29 (28%)	0.069
**Transferred from another hospital**	50 (46%)	48 (46%)	0.999
**Arrival via ambulance**	64 (59%)	48 (46%)	0.076

CAD, coronary artery disease

Unstable vital signs: systolic blood pressure<90 mmHg, heart rate<50/min, intubated, out of hospital cardiac arrest, or under intravenous vasopressor or inotropic agents.

### Door-to-ECG time

**Fig 4:**
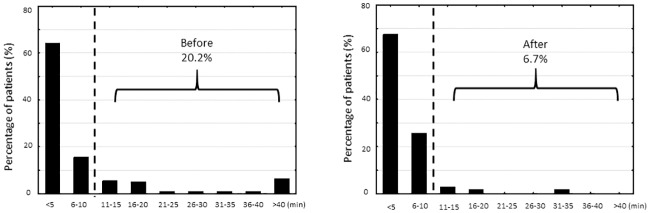
Distribution of door-to-ECG times before and after intervention.

The distribution of door-to-ECG times before and after intervention is displayed in Fig 4. The intervention group had significantly higher rates of meeting the door-to-ECG target time of within 10 minutes than did the control group: 98 of the 105 patients (93.3%) in the intervention group and 87 of the 109 patients (79.8%) in the control group. No significant difference in door-to-ECG time was observed: 5 minutes (IQR = 3–6 minutes) in the intervention group and 4 minutes (IQR = 2–7) in the control group. (Table 2) The improvement in the rate of meeting the door-to-ECG target time was observed in the subgroup of non-transferred STEMI (91.2% vs. 71.1%, *p* = 0.008) and in the subgroup of walk-in patients with STEMI (91.2% vs. 73.3%, *p* = 0.030). In univariate analysis, male sex, increase in age, smoking history, arrival by ambulance, transferred patients, and the quality-improving interventions were associated with the attainment of door-to-ECG target time. After adjustment for co-variates including age, sex, risk factors, unstable vital signs, and mode of arrival, the quality-improving interventions remained a significant contributing factor for achieving door-to-ECG time within 10 minutes. (Table 3).

**Table 2:** Median times and rates of meeting door-to-ECG target time and door-to balloon time before and after intervention.

Outcome	Median time (min)		*p-value*	Rate of meeting target time (%)		*p-value*
	Before	After		Before	After	
**Door-to-ECG (N)**	109	105		109	105	
**Overall**	4 (2–7)	5 (3–6)	0.963	87 (79.8%)	98 (93.3%)	0.005
**Transferred from another hospital**						
No	4 (1–15)	5 (3–7)	0.777	42 (71.1%)	52 (91.2%)	0.008
Yes	3 (1–5)	4 (2–6)	0.186	48 (96.0%)	47 (97.9%)	0.999
Mode of arrival						
Walk-in	5 (2–14)	5 (3–7)	0.860	33 (73.3%)	52 (91.2%)	0.030
Ambulance	4 (2–6)	4 (2–6)	0.689	57 (89.1%)	47 (97.9%)	0.135
**Door-to-balloon (N)**	101	101		101	101	
**Overall**	66 (51–90)	65 (50–78)	0.054	77 (76.2%)	92 (91.1%)	0.007
**Transferred from another hospital**						
No	86 (69–126)	66 (60–83)	<0.01	32 (60.4%)	44 (83.0%)	0.017
Yes	53 (43–64)	47 (53–72)	0.080	45 (93.8%)	48 (100.0%)	0.242
**Mode of arrival**						
Walk-in	85 (68–107)	66 (60–83)	<0.01	28 (68.3%)	44 (83.0%)	<0.001
Ambulance	54 (44–81)	53 (47–72)	0.616	49 (81.7%)	48 (100.0%)	0.140

Time: median (interquartile range, IQR); Target: <10 min for door-to-ECG time; <90 min for door-to-balloon time.

ECG = electrocardiogram.

**Table 3:** Univariable and multivariable analysis for predictors of door-to-ECG time <10 min and door-to-balloon time <90 min.

Factors	Univariate	*p-value*	Multivariate	*p-value*
**Door-to-ECG less than 10 minutes**				
Male sex	3.14 (1.39–7.01)	0.006	1.43 (0.49–4.20)	0.517
Age	0.96 (0.93–0.99)	0.006	0.98 (0.94–1.01)	0.223
Hypertension	0.71 (0.31–1.64)	0.471	0.96 (0.36–2.60)	0.939
Diabetes	0.72 (0.32–1.58)	0.406	0.66 (0.26–1.69)	0.385
Smoker	4.26 (1.43–12.7)	0.010	2.69 (0.76–9.51)	0.125
Hyperlipidemia	1.89 (0.82–4.37)	0.137	2.27 (0.80–6.38)	0.122
CAD history	1.55 (0.44–5.45)	0.498	2.16 (0.51–9.25)	0.299
Unstable vital signs	0.87 (0.35–2.18)	0.761	0.44 (0.13–1.45)	0.178
Ambulance	2.34 (1.03–5.30)	0.042	1.02 (0.25–4.17)	0.980
Transferred	4.85 (1.78–13.2)	0.002	5.65 (1.18–27.1)	0.030
QI initiatives	3.54 (1.44–8.69)	0.006	3.83 (1.32–11.1)	0.013
**Door-to-balloon time less than 90 minutes**				
Male sex	1.78 (0.79–4.01)	0.162	0.91 (0.28–2.92)	0.872
Age	0.98 (0.95–1.00)	0.092	0.99 (0.95–1.02)	0.423
Hypertension	0.80 (0.36–1.76)	0.579	0.78 (0.30–2.03)	0.614
Diabetes	0.99 (0.46–2.15)	0.971	0.77 (0.30–1.99)	0.773
Smoker	1.80 (0.79–4.01)	0.165	1.28 (0.42–3.85)	0.664
Hyperlipidemia	1.76 (0.80–3.84)	0.160	1.82 (0.67–4.90)	0.237
CAD history	0.67 (0.13–3.37)	0.670	0.89 (0.25–3.23)	0.861
Unstable vital signs	1.08 (0.43–2.68)	0.872	0.77 (0.22–2.67)	0.772
Ambulance	2.69 (1.23–5.90)	0.013	0.28 (0.07–1.21)	0.089
Transferred	12.2 (3.59–41.7)	<0.001	40.2 (7.71–276)	<0.001
QI initiatives	3.19 (1.39–7.26)	0.006	2.96 (1.09–8.03)	0.033

CAD, coronary artery disease; QI, quality-improving; ECG = electrocardiogram; Unstable vital signs: systolic blood pressure<90 mmHg, heart rate<50/min, intubated, out of hospital cardiac arrest, or under intravenous vasopressor or inotropic agents.

### Door-to-balloon time

A total of 202 patients underwent primary PCI for STEMI: 101 patients before and after intervention, respectively. No significant difference in the median door-to-balloon time between the control (median, 64 min; IQR, 51–83 min) and the intervention group (median, 65 min; IQR, 50–78 min) was observed. Nonetheless, the intervention group had a higher rate of meeting the door-to-balloon target time than the control group (91.1% vs. 76.5%, *p* = 0.007). The improvement in the rate of meeting the door-to-balloon target time was observed in the subgroup of non-transferred STEMI (83.0% vs. 60.4%, *p* = 0.017) and in the subgroup of walk-in patients with STEMI (83% vs. 68.3%, *p*<0.001) (Table 2). In univariate analysis, arrival by ambulance, transferred patients, and the quality-improving interventions were associated with the attainment of door-to-balloon target time. After adjustment for co-variates, including age, sex, risk factors, unstable vital signs, and mode of arrival, the quality-improving interventions remained a significant contributing factor for achieving door-to-balloon time within 90 min. (Table 3).

### The impact and contributors of door-to-ECG times

Before the intervention, the door-to-ECG interval had the highest incidence of delay (20.8%), followed by the ECG-to-activation interval (9.9%), activation to catheterization laboratory interval (2.0%), and catheterization laboratory to balloon interval (2.0%). After the quality-improving initiative, the most common interval for delay was still the door-to-ECG interval, but the incidence decreased to only 6.7% (Table 4).

**Table 4:** Time intervals and causes of delay for door-to-balloon time before and after quality-improving initiatives.

Components of times (threshold) and percentage with causes of delay	Before (N = 101)	After (N = 101)
**Door-to-ECG (<10 min)**	**21 (20.8%)**	**6 (5.9%)**
Unrecognized symptoms	14 (13.9%)	3 (3.0%)
Resuscitation	3 (3.0%)	2 (2.0%)
Delay in performing ECG	4 (4.0%)	3 (3.0%)
**ECG-to-Activation (<20 min)**	**10 (9.9%)**	**7 (6.9%)**
Interpretation of ECG	4 (4.0%)	3 (3.0%)
Resuscitation	3 (3.0%)	2 (2.0%)
Additional diagnostic study	1 (1.0%)	1 (1.0%)
Consent issues	1 (1.0%)	2 (2.0%)
**Activation-to-Cath Lab (<30 min)**	**2 (2.0%)**	**1 (1.0%)**
Cath team arrival	1 (1.0%)	0 (0%)
Transportation	1 (1.0%)	1 (1.0%)
**Cath Lab-to-Balloon (<30 min)**	**2 (2.0%)**	**2 (2.0%)**
Resuscitation	0 (0%)	1 (1.0%)
Difficult procedure	2 (2.0%)	1 (1.0%)
**Door-to-Balloon (90 min)** *	**25(24.8%)**	**9 (8.9%)**

*: A patient may have more than one phase or cause of delay.

ECG, electrocardiogram; Cath, catheterization.

The most common cause of door-to-balloon delay before intervention was unrecognized symptoms (13.9%), followed by delayed interpretation of the ECG (4.0%), delay in performing the ECG (4.0%), and resuscitation (3.0%). After the intervention, the most significant difference arose from a decrease in the proportion of delays caused by unrecognized symptoms (from 13.9% to 3.0%) (Table 4).

**Fig 5:**
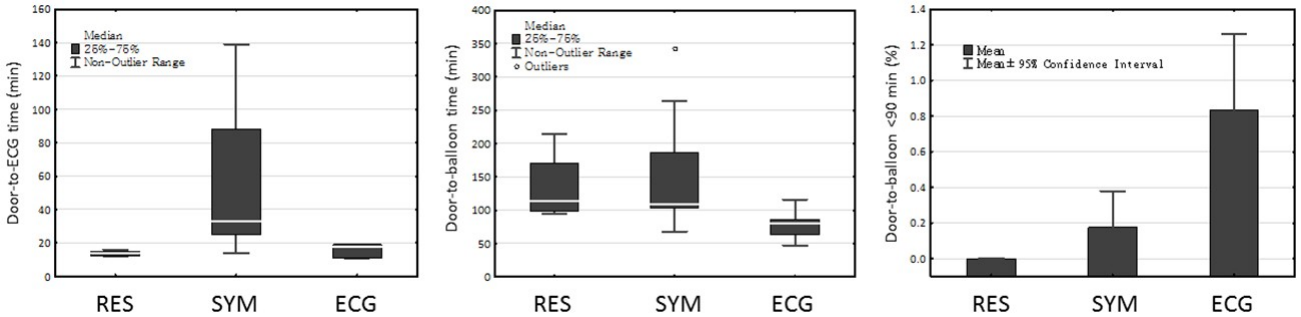
The impact of different contributors to door-to-ECG delays on door-to-ECG times, door-to-balloon times, and percentage of meeting the door-to-balloon target time of <90 minutes. (Abbreviations: RES, resuscitation; SYM, unrecognized symptoms; ECG, delay in performing the ECG).

When the outliers of door-to-ECG times were stratified by their causes, the group of unrecognized symptoms had both longer median time and wider variation of door-to-ECG time, compared to that caused by resuscitation or performing ECG. (Fig 5) The median door-to-balloon times of patients with unrecognized symptoms or resuscitation were both above the 90 min target, but not the group due to delayed performance of the ECG. Most of the patients who were delayed due to resuscitation and unrecognized symptoms failed the 90 min door-to-balloon target. (Fig 5).

### The cost for improving door-to-ECG times

In the first year after interventions, the number of ECGs performed in the ED was 2067 per month (census of ED visits, 6110 per month; 0.338 ECG per visit), 31% more than that in the year before interventions (1517 ECGs per month; census of ED visits, 5859 per month; 0.259 ECG per visit). After adjusting for the census of ED visits, additional 485 ECGs (6110–6110 x 0.259) were performed per month after the quality-improving initiative. Because no facility or personnel was added for the initiative, the increase in cost was estimated to be 2425 US dollar per month (5 US dollar per ECG examination in our country). Based on the improvement of door-to-ECG target rate by 14% (from 79% to 93%), an additional 14.7 patients (105 x 14%) achieved door-to-ECG target in the first year after intervention. Therefore, the cost to increase one patient achieving door-to-ECG time target would be 1980 US dollars.

## Discussion

### Brief summary of the findings

In this study we found that the contribution of door-to-ECG times in door-to-balloon times increased after implementation of best practice techniques for door-to-balloon times recommended by the guidelines. We could also optimize the rate of meeting the door-to-ECG target time through modification of the triage process. Finally, the optimization of the door-to-ECG process further improves the rate of meeting the door-to-balloon target time, especially for patients who are not transferred from another hospital.

### Impact of door-to-ECG time

National guidelines have focused on the adoption of best practice techniques to improve door-to-balloon times. Although door-to-balloon times are decreasing, current techniques focus on the processes after STEMI diagnosis. Nonetheless, the processes before the diagnosis of STEMI were also relevant determinants of the door-to-balloon times [17]. Even after the implementation of best practice techniques suggested by the door-to-balloon Alliance, door-to-activation times remained a key determinant of overall door-to-balloon times, and were highly variable, as demonstrated by the Activate-SF registry [18]. Our study provided novel information because the components and contribution factors of door-to-activation times were parsed out for analysis. Our data suggested that the contribution of processes after the STEMI is recognized, such as arrival of the cardiac catheterization team, transportation to the catheterization lab, and the process of intervention lessened. In contrast, the process before the STEMI is recognized, especially door-to-ECG time, became more paramount for the door-to-balloon process.

Although there is a relative lack of research on door-to-ECG times, data from previous studies suggested that the triage process in the ED is critically important. In a study of patients with STEMI at triage, one-fourth of the patients with STEMI were placed in the low-priority group, which was associated with significant delay in door-to-ECG and door-to-balloon times [18]. Our data identified that atypical symptoms, such as shortness of breath, syncope, weakness, and gastrointestinal discomfort, were the main sources of delay in door-to-ECG times. Moreover, the impact of identification failure on door-to-balloon times was more prominent than delay in performing the ECGs. When a STEMI was not recognized in the triage stage, the average time to diagnosis was usually more than 30 min, making the door-to-balloon threshold difficult to achieve. In contrast, the delay in time due to performing the ECG was usually less than 5 min, which could be compensated for in the subsequent processes. Our results were supported by previous studies that demonstrated that atypical symptoms are an important cause of delayed reperfusion [19–21]. Accordingly, after the implementation of best practice techniques suggested by the door-to-balloon Alliance, a reliable process to recognize patients with STEMI at triage becomes paramount for timely reperfusion.

### Strategies to improve door-to-ECG time

The sources of delays in door-to-ECG times could be classified into 2 types: failed recognition or delayed performance. According to the review of our cases, the majority of door-to-ECG delays came from failed recognition [21]. Although it is intuitive to obtain ECGs for all symptoms possible for coronary ischemia, STEMI being missed was common for the one-third of patients without chest pain [22]. Before the modification of our ECG process, performing ECG at triage depended on the judgement of the triage nurse. Despite the knowledge of ischemic symptoms other than chest pain, their judgement was still biased by the appearance of the patients and the severity of their symptoms. Therefore, failed recognition still occurred, especially in patients with symptoms other than chest pain. After strict implementation of a protocol-driven ECG in the triage process, less patients with STEMI were missed due to failed recognition. This measure was supported by similar protocol-driven triage processes in previous studies [23–25].

Before the initiative, the delay in performing an ECG usually occurred when the ECG technician had other work at hand. It might also have occurred when the ED was overcrowded, or the ECG area was occupied. To further expedite the door-to-ECG process, an additional ECG machine and a pre-specified bed for ECG was set up near the triage area. This modification expedited the ECG process. Furthermore, it created a friendly setting that made assistance by the triage nurse easier.

Strategies in previous studies focused on the identification of eligible patients at the door. Usually, an extra staff member, a greeter, or a trained registration clerk was needed for the assessment of patients at arrival [12–14]. Different from previous studies, we focused on the standardization of the criteria to initiate an immediate ECG at the triage stage. A major concern of this strategy was an increase in the number of ECGs in the ED. In addition, the ECG technicians might be overworked not only because of the increase in the number of ECGs to be performed but also the urgency under the new protocol. We found a 30% increase in the number of ECGs after the protocol-driven ECG process was applied. The extra load on the ECG technicians could be lessened by the assistance of the triage nurse and no increase in manpower will be needed. Nonetheless, a detailed cost-effectiveness analysis for this approach deserves further evaluation.

### Improving target rate of door-to-balloon times

Although the national guidelines recommended a door-to-ECG time of less than 10 minutes, the impact of door-to-ECG time on outcomes or on door-to-balloon time of patients with STEMI has seldom been addressed. Multiple strategies were usually used in previous studies to the effect that individual techniques were hard to quantify. Furthermore, their effects on door-to-balloon times were rarely reported. Among these studies of door-to-ECG times, only one study demonstrated a parallel decrease in door-to-balloon times. Our study was unique because it was conducted under the implementation of best practice techniques suggested by the door-to-balloon Alliance. We demonstrated that failed recognition at the triage stage played a more prominent role. Furthermore, we demonstrated a corresponding improvement in the rate of meeting the door-to-balloon target time.

Nonetheless, the benefit on door-to-balloon time was not observed in the subgroup of patients who were transferred from another hospital. These patients usually already have had an ECG performed to establish diagnosis prior to transfer. The pre-hospital transmission of ECGs or diagnosis would reduce the relevance of door-to-ECG process on door-to-balloon times. The benefit on door-to-balloon time was also absent in the subgroup of patients who were transferred by ambulance. Previous studies have demonstrated that the utilization of emergency medical service (EMS) among STEMI patients was associated with faster symptom-to-balloon times [9,26]. Nonetheless, in our country and other Asia countries, a substantial proportion of STEMI patients were not transferred from another hospital or EMS [7,27,28]. Therefore, our data suggest that continued emphasize on the triage process of walk-in patients is paramount for a timely reperfusion therapy.

### Limitations

Some limitations should be addressed. First, this study was performed at a single center in an urban environment and therefore may lack external validity. Second, for a before-and-after study design, there may be unmeasured confounders that could not be adjusted in multivariate analysis. The evidence is less robust compared with that generated from randomized controlled trials. Although we have analyzed the increase in the number of ECGs before and after the interventions, a more comprehensive cost-effective analysis is needed for this protocol-driven ECG initiation process.

### Conclusions

Although further effort to reduce door-to-balloon time below the 90-minutes target may not reduce mortality, the value to minimize outliers of the 90-minute target cannot be denied. After implementation of the best practice techniques suggested by the door-to-balloon Alliance, early recognition of patients with STEMI was paramount for the optimization of patients achieving the 90-minute target. A multidisciplinary approach, especially with protocol-driven ECG initiation at triage, could decrease the outliers of door-to-ECG times with a corresponding decrease in outliers of door-to-balloon times, especially for patients who are not transferred from another hospital.
